# Automated decision support in melanocytic lesion management

**DOI:** 10.1371/journal.pone.0203459

**Published:** 2018-09-07

**Authors:** Stephen J. Gilmore

**Affiliations:** 1 Skin and Cancer Foundation, Melbourne, Australia; 2 Dermatology Research Centre, Diamantina Institute, University of Queensland, Brisbane, Australia; Liverpool John Moores University, UNITED KINGDOM

## Abstract

An automated melanocytic lesion image-analysis algorithm is described that aims to reproduce the decision-making of a dermatologist. The utility of the algorithm lies in its ability to identify lesions requiring excision from lesions not requiring excision. Using only wavelet coefficients as features, and testing three different machine learning algorithms, a cohort of 250 images of pigmented lesions is classified based on expert dermatologists’ recommendations of either excision (165 images) or no excision (85 images). It is shown that the best algorithm utilises the Shannon4 wavelet coupled to the support vector machine, where the latter is used as the classifier. In this case the algorithm, utilising only 22 othogonal features, achieves a 10-fold cross validation sensitivity and specificity of 0.96 and 0.87, resulting in a diagnostic-odds ratio of 261. The advantages of this method over diagnostic algorithms–which make a melanoma/no melanoma decision–are twofold: first, by reproducing the decision-making of a dermatologist, the average number of lesions excised per melanoma among practioners in general can be reduced without compromising the detection of melanoma; and second, the intractable problem of clinically differentiating between many atypical dysplastic naevi and melanoma is avoided. Since many atypical naevi that require excision on clinical grounds will not be melanoma, the algorithm–in contrast to diagnostic algorithms–can aim for perfect specificities without clinical concerns, thus lowering the excision rate of non-melanoma. Finally, the algorithm has been implemented as a smart phone application to investigate its utility in clinical practice and to streamline the assimilation of hitherto unseen tested images into the training set.

## Introduction

The incidence of melanoma has increased substantially in the United States, Europe and Australia over the last 30 years [1]. While incidence rates are projected to increase in both the United States and Europe over the next two decades, they are expected to stabilise in Australia [1]. Stabilisation of melanoma rates in Australia is largely thought to be a consequence of the public awareness campaigns that began in the early 1980s. Increased physician diagnostic vigilance and increased public awareness of melanoma–due to the aforementioned public awareness campaigns–has, however, led to large increases in office surgery, where the majority of pigmented lesions excised are not melanomas [2]. This phenomenon can be captured by a measure known as the ‘*Number Needed to Treat*’ (NNT), a term loosely defined as the number of benign lesions excised per melanoma [3]. There exists a trade-off here–if the NNT is too high, then many lesions are excised unnecessarily; in contrast, a low NNT suggests melanomas may be missed. Too many excisions increases patient morbidity, can lead to problems associated with over-diagnosis [4], and will be associated with ballooning publicly funded health care costs. On the other hand, a low NNT may imply a potential for increased mortality.

The considerations above raise the issue of what value an optimal NNT should assume. For non-dermatologists, reported values include 19.6, 23, 22 and 30 [5–8]. In contrast, the NNT for dermatologists may be lower–values of 6.3 and 6.5 have been reported [9,10]. If it is accepted that dermatologists have greater diagnostic acumen than non-dermatologists in the clinical asessment of melanocytic lesions, then it is likely that the former are not missing more melanomas in comparison with the latter, despite their lower NNTs. How, then, can the average NNT, with respect to all practioners, be lowered without compromising melanoma detection? Evidence suggests further training of primary care physicians can increase the yield of melanoma as a proportion of all excisions [11]. But the impact of further training will be limited if it is not sought by the majority of practitioners. On the other hand, the utilisation of machine learning in the clinical setting by non-specialists has the potential to lower the NNT without requiring practitoners to acquire additional skills. Importantly, it has been shown that practitioners are willing to change their decision with respecct to melanocytic lesion management if supplied with a machine learning decision [12]. Results from a large prospective clinical trial demonstrate the utility and posible limitations of algorithm-based decision support in melanoma diagnosis [13].

There exists a large literature regarding machine learning and melanocytic lesion assessment [14–17]. Nearly all melanocytic lesion classification schemes reported thus far use melanoma, histologically diagnosed, as a unitary class in classification.^.^ Such algorithms–which can be labelled *diagnostic algorithms–*are designed to distinguish melanoma from non-melanoma and are thus in effect making a diagnosis. However, these approaches are not without shortcomings, three of which are briefly considered here. First, there may exist training inaccuracies due to the lack of consistency among pathologists regarding the diagnosis of atypical lesions [18]. Second, the task of reliably differentiating between many atypical dysplastic naevi and melanoma, on macroscopic morphologic grounds, is likely to be an intractable problem. And finally, from the management perspective, the non-expert may be better served by knowing whether a lesion in question should be excised, not whether it is melanoma or non-melanoma. Consider a severely dysplastic naevus that a diagnostic algorithm correctly classifies as non-melanoma. In this instance, the dermatologist is likely to excise the lesion despite correctly favouring non-melanoma (if asked) as the diagnosis (to exclude the low, but nonetheless significant possibility of melanoma). Such a scenario will create the undesirable outcome of discord between the management decision of the dermatologist and non-specialist.

**Fig 1:**
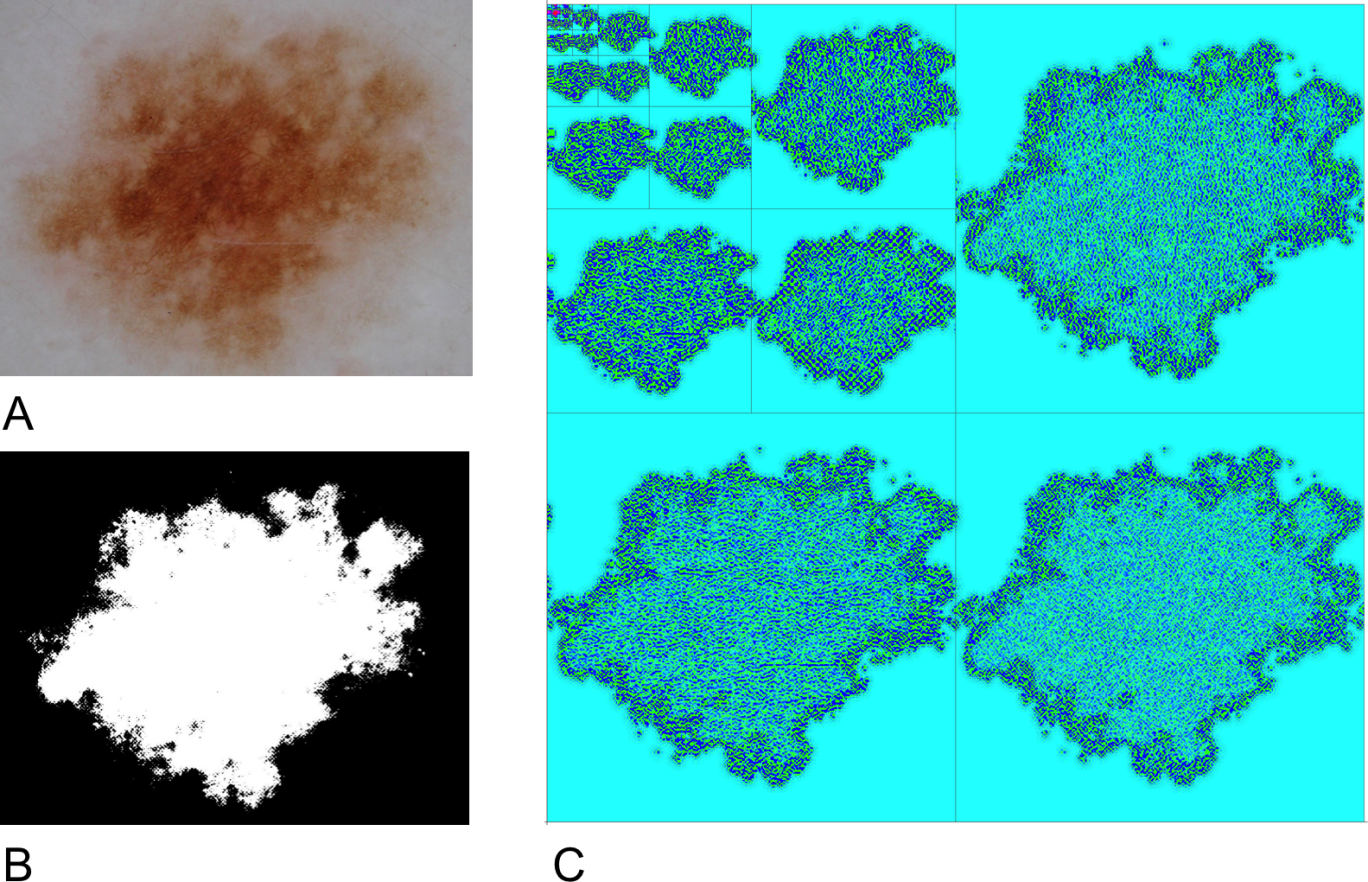
Image segmentation and wavelet decomposition. **(A)** Benign naevus. Note the global asymmetry and irregular and poorly-defined borders. **(B)** Chan-Vese segmentation of this lesion. **(C)** Graphical representation of a six-level wavelet decomposition of the naevus shown in **(A)**. Wavelet coefficients are reprented as a color-coded grid as follows: HH (lower-right); HL (upper right) and (LH) lower-left. The LL band is similarly decomposed as per the upper left square. This process is iterated six times.

Much of the difficulty in distinguishing between atypical naevi and melanoma arises because the diagnostic morphologic features that are routinely used and detected by computer algorithms (and clinicians) are never entirely specific for benign or malignant lesions. For example, while ‘suspicious’ features such as asymmetry or poorly defined boundaries are commonly present in benign naevi (see Fig 1A), suspicious ‘melanoma-specific’ features such as irregular globules may be present in atypical naevi. Many melanomas will be less atypical than some atypical dysplastic naevi. In practice, however, by utilising multiple features, and applying a non-linear classification scheme–such as a support vector machine–these problems can be mitigated. In some cases, algorithms may yield a probability of melanoma, requiring interpretation by the clinician. Binary decision difficulties can be partially overcome by placing greater weight on sensitivity, with a reduction in the false negative rate. However, increased sensitivity will result in decreased specificity. Yet it is often considered desirable for diagnostic algorithms to have a ‘healthy’ false positive rate–this stems from the tacit assumption, noted above, that it is likely to be impossible, in principle, to distinguish, on clinical grounds, between many cases of atypical dysplastic naevi and melanoma. Colloqually speaking, the algorithm may need to get it wrong to get it right.

Although the considerations above give pause to the idea of utilising diagnostic algorithms in automated melanocytic assessment, they are nonetheless valuable and represent the state of the art. Indeed, recent work published in *Nature* describes a deep convoluted neural network trained to make a diagnosis of melanocytic (and other) dermatologic lesions based on tens of thousands of training images, highlighting both the relevance of diagnostic algorithms, and the value of brute-force computation [19]. However, the shortcomings noted above with respect to diagnostic algorithms suggest that it may be worthwhile investigating the possibility of an alternative approach: the development of a *decision-making* algorithm. In this case, the label ‘decision-making’ is applied since such an algorithm will simply determine whether a melanocytic lesion should be excised or not excised. The training set will be partitioned based on expert dermatologists’ assessment regarding excision/no excision, rather than on histopathological diagnoses, hence the algorithm will replicate decision-making expertise. Importantly, there is no need for the false positive rate to be significantly less than 1; the algorithm can operate with specificities approaching 1 without any clinical concerns.

But should the feature set used by a decision-making algorithm be the same as that used for a diagnostic algorithm? Although there will exist considerable overlap between any set of morphologic criteria that identifies melanoma and identifies lesions requiring excision (after all, the purpose of clinical evaluation of melanocytic lesions is to identify potential melanoma), the feature set cannot be the same with respect to the classification problem because the respective algorithms are classifying *different* classes of objects. If the same set of features are applied to the classification of different classes of objects, then one or the other will be suboptimal. While these observations do not prove that a feature set derived using well-defined morphologic parameters could not be an efficient classifier with respect to a decision-making algorithm, it is nonetheless apparent that feature selection would involve considerable subjectivity, and require multiple rounds of training and testing.

Motivated by these considerations, an alternative approach will be implemented: the feature selection process desribed here will be limited to obtaining and analysing the statistical properties of *wavelet coefficients* derived from dermoscopic image data of melanocytic lesions. The feature selection process does not then explicitly characterise well-defined morphologic parameters (for example, the blue-grey veil) and thus reduces the subjectivity associated with generating a feature selection list that may need to be different from that associated with a diagnostic algorithm. Wavelet coefficients encode textural information at different length-scales and are thus well-suited to the analysis of pigmented melanocytic lesion image data given the fractal structure of the latter [20]. This approach is also partially motivated by the idea that an experienced dermatologist will *know* when a lesion requires excision, but it may not be possble for he or she to precisely specify, with a list of well-defined computer-recognisable features, why this is so. In contrast, defining a list of features that (imperfectly) characterise melanoma is a relatively straightforward task.

In the following sections it wil be shown that the use of wavelet coefficients *per se* does not compromise accuracy: high sensitivities and specificities, approaching the expertise of dermatologists, can be achieved. If machine intelligence can reproduce the decision-making of a dermatologist, then the average NNT of practitioners in general will be lowered. Here it is proposed that the algorithm should act as a *decision support tool* [21]; that is, the clinician should use any other external information about any lesion under consideration as deemed relevant, such as size, site, history and context, in order to arrive at a management decision. The following sections describe the approach to this problem, the results, and discuss the utility of the algorithm–which has been developed as a smart-phone application for research purposes–in the clinical setting.

## Methods and results

### Database

Two hundred and fifty polarised dermoscopic images were obtained from the Department of Dermatology at the Medical University of Graz in Austria over the period 2003–2008. Digital photographs were taken using a DermLite FOTO lens (3Gen LLC; Dana Point, CA, USA) coupled to a 4500 CoolPix digital camera (Nikon Corporation, Tokyo, Japan) without flash using the camera’s auto setting. Eighty-five lesions were considered benign by an expert dermatologist and were not excised. Note, however, that a benign diagnosis does not always imply that the lesion is trivially bland (Fig 1A). All remaining lesions were considered atypical enough to warrant excision and were subsequently examined microscopically by expert dermatologists using standard diagnostic criteria. Eighty-five of these lesions were diagnosed as melanoma while eighty lesions were diagnosed as dysplastic naevi.

### Image pre-processing

All images were rescaled to 1000 pixels wide in the maximal dimension. Lesion segmentation was performed using a two-level Chan-Vese algorithm [22] (Fig 1B), which, in the current implementation, can operate on color images [23]. The Chan-Vese algorithm iteratively minimises the functional

$$f\left(c_{1},c_{2},F\right)=\mu_{1}{\left(\varphi \right)}_{L}+\mu_{2}{\left(D\right)}_{A}+\lambda_{1}\iint_{D}{\left|F-c_{1}\right|}^{2}dxdy+\lambda_{2}\iint_{\Omega /D}{\left|F-c_{2}\right|}^{2}dxdy$$

where *f* is parametrized by the length penalty *μ*_1_, the area penalty *μ*_2_ and the level penalties *λ*_1_ and *λ*_2_. The total image region Ω is divided into 2 segments *D* and Ω/D with contour Ψ=ðD, while *c*_1_ and *c*_2_ are constants given by the mean of *F* in *D* and the mean of *F* in Ω/D respectively. The values of all adjustable parameters were used at their default settings [23].

A bounding box was then drawn at the lesion extremities and all images, using blank pixels, were extended in the shortest dimension to form a square such that the final dimensions were 2^9^ x 2^9^ = 512 x 512 pixels. No other pre-processing steps, such as histogram normalisation, filtering, or removal of artefacts, were undertaken prior to analysis. Severely compromised images, such as those exhibiting excessive hair or prominent specular reflections from scales, were excluded from the database.

### Wavelets

Wavelets are short wave-like functions that can represent any signal, including time series or image data, by appropriate scaling and translation. They have been previously used, with some success, in the automated diagnosis of melanoma [24–26]. As noted in the Introduction, a wavelet decomposition is a numeric representation of an image at different scales–its spectral properties. The tree of wavelet coefficients at level ***j*** comprises coarse coefficients *c* given by the forward transform

$$c_{j+1,n}=\sqrt{2}\sum_{m}a_{m-2n+2}c_{j,m}$$

and fined-grained coefficients *d* given by the forward transform

$$d_{j+1,n}=\sqrt{2}\sum_{m}b_{m-2n+2}c_{j,m}$$

where the *c*_0,*n*_ represent original image data and the *a*_*i*_ and *b*_i_ are the low and high-pass filter coefficients respectively. A six-level wavelet decomposition of all images was performed using the Shannon4 discrete wavelet transformation (Fig 1C). Each decomposition level yields 4 frequency sub-bands (High-High, High-Low, Low-High and Low-Low), calculated with respect to pixel values associated with each of the RGB colour channels and the luminance channel.

For each channel and for each sub-band, four statistical measures (the mean, the absolute mean, the energy and the variance) was measured. For each channel and sub-band the mean and the variance was calculated with respect to the skewness and kurtosis, where for the latter each calculation is derived from each single array of pixels in both the vertical and horizontal directions. This procedure thus generated 6 (decompositions) x 4 (sub-bands) x 4 (channels) x 12 (4 global measures and 4 x 2 axis-specific measures) = 1152 feature values per image. These data are available as Supporting Information files.

### Feature selection

**Fig 2:**
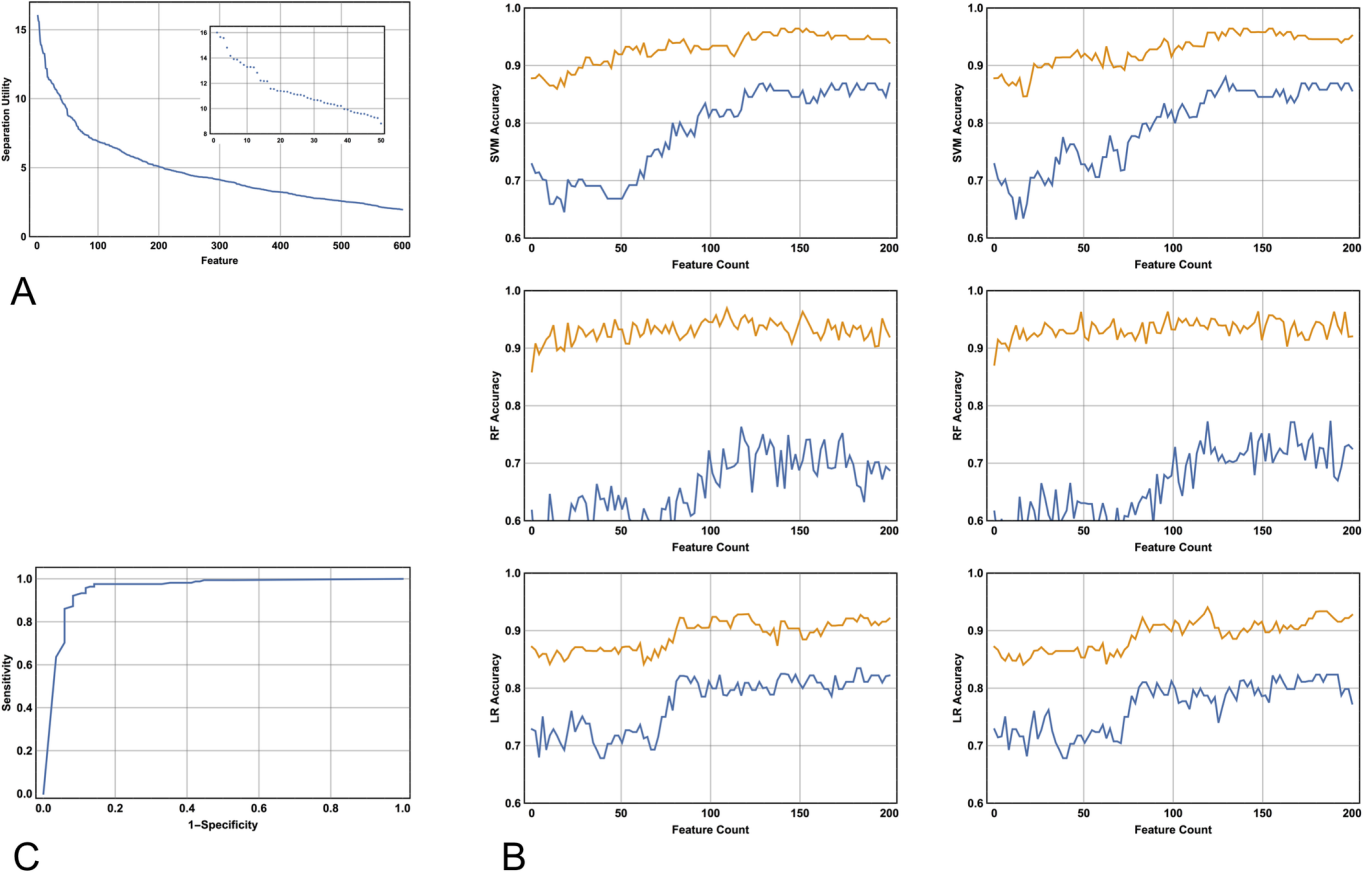
The ReiefF algorithm, model sensitivity and specificity and the receiver operated characteristic (ROC) curve. **(A)** Running the ReliefF algorithm yields an ordered list of features that best separate the classes. Here the best 600 features are plotted against their utility in separation. The first 50 feature values are shown in detail (Inset). Note that the major inflection point occurs at approximately feature 125. **(B)** Ten-fold cross-validation sensitivity (mustard) and specificity (blue) as a function of the number of first *N* features used in the model. Of all parameter choices (see Table 1), the two best performed SVM, RF and LR models are shown in the first, second and third rows respectively. Note that the SVM outperforms the RF and LR models, and note that incorporating additional features beyond 125 does not improve performance, an observation that is independent of the choice of algorithm. **(C)** Leave-one-out cross validation ROC curve utilising the Shannon4 wavelet decomposition and the SVM. The SVM outputs a probability of diagnosis with respect to the classes. The upper left of the curve reveals two important points dependent on the decision probability cutoff: a sensitivity of 0.97 and a specificity of 0.89; and a sensitivity of 0.93 and a specificity of 0.93. Both points are equally accurate, but the latter is closer to the point (0,1).

To rank the features in their ability to separate the classes (which are lesions excised versus lesions not excised) the ReliefF algorithm was employed [27]. ReliefF is a reliable and widely used algorithm with respect to the analysis of microarray data [28]. By analogy, it is therefore a suitable choice for melanocytic lesion classification: the bioinformatics problem often involves finding a set of genes that best distinguish two classes (for example, good versus poor-prognosis subsets). In addition, the data matrix of a typical microarray result has an identical structure to the wavelet coefficient matrix. The methodology proceeds in an iterative manner by measuring distances between data points, which, in this case, are embedded in a 1152 dimensional feature space. The output converges to an ordered ranking of features that best separate the classes (Fig 2A). Choosing the optimal combination of features from an ordered list of length *N* that best separates the classes is, in general, an intractable problem since the solution may not necessarily include *all* of the first *n* features, where *n* is an integer between 1 and *N*. The feature selection problem is addressed here as follows: first, the cumulative feature count of the ordered list obtained from running the ReliefF algorithm is plotted against performance (here the performance is given by the mean of the sensitivity and specificity; and where the aforementioned metrics are calculated with respect to 10-fold cross-valdation). Second, local maxima, if present, will be identified. It will be shown below that these plots exhibit a threshold number of features *n* such that classification performance is not improved for feature counts greater than *n*. This finding indicates that it is possible to calculate the minimum number of features that *add* information to the class separating task.

### Machine intelligence

Three algorithms were investigated in terms of their utility in modelling the class separation: the SVM [29, 30], the random forest algorithm (RF) [31] and logistic regression (LR) [32]. All algorithms were investigated utilising the Shannon4 wavelet decomposition. Optimisation of the classification task was performed by investigating parameter space: this was achieved by varying the value of C with respect to the SVM; by varying the number of trees $N_{tr}$ with respect to the RF algorithm; and finally, by varying either the L1or L2regularisation parameters with respect to the LR algorithm. For each algorithm 10-fold cross validation was performed with respect to the number of selected top ranked features, ranging from 1 to 200 [23]. These results are shown in Table 1 and Fig 2B. Note that the SVM (briefly desribed below) yields the most accurate result, and that the value of nis around 125 (Fig 2B); this latter value is largely independent of the algorithm used in the analysis.

**Table 1:** Classifier-dependent 10-fold cross-validation error rates.

**SVM**						
C	**0.5**	**2**	**10**	**30**	**40**	**50**
Error	0.114	0.107	0.086	0.087	0.084	0.087
**RF**						
No. of trees	**80**	**120**	**160**	**200**	**240**	**280**
Error	0.142	0.141	0.146	0.145	0.144	0.143
**LR**						
Reg. Param.	**0.001 (L1)**	**0.01 (L1)**	**0.1 (L1)**	**0.001 (L2)**	**0.01(L2)**	**0.1 (L2)**
Error	0.138	0.133	0.130	0.127	0.121	0.119

Best-performing ten-fold cross validation models (of all models with feature counts between 1 to 200) for each classification algorithm and for different parameter values. For the SVM, the value of the gamma scaling parameter is optimised at 0.007. For the random forest model, the leaf size is optimised at 1 for any number of trees. Note that for the logistic regression model L2regularisation outperforms L1regularisation, but does not achieve the accuracy of the SVM, even for poor choices of ***C***.

### Support vector machines

Given that the SVM achieves the best performance with respect to the calculations described above, the RF and LR models will not be pursued. These results, although not exhuastive, are consistent with findings reported elsewhere: among all kernel-based methods, it is widely recognised that SVMs are likely to yield the best performance [33].

Developed by Vatnik [29], SVMs originated within the field known as statistical learning theory, where the objective is to minimise the risk, or generalisation error, of the model. Briefly, the SVM provides a decision function gwhich is the optimal solution to a quadratic programming problem subject to constraints [30]. The quadratic programming problem incorporates a parameter ***C***: its value quantifies the trade-off between the width of the margin separating the classes and classification error. For all subsequent SVM calculations, the kernel Kis given by the Gaussian radial basis function

$$K\left(x,x^{|}\right)=Exp\frac{1}{\sigma }\left[-\left(x-x^{|}\right)\cdot \left(x-x^{|}\right)\right]$$

where σ is an adjustable parameter. The output of the SVM provides a probability of class membership; for all binary decisions reported above and below a cut-off of 0.5 was used. Current applications, for example, include computational biology [34, 35], and in the classification of melanocytic lesions [21, 36].

### Model accuracy

To further characterise the SVM, 10-fold, 25-fold and leave-one-out cross validation was performed for six different values of C,repeating the cross validation 50 times for each combination of Cand level of cross validation [37]. These results are shown in Table 1. Note how the model accuracy improves as the training set increases in size, and note how the optimal value of C is 30. The best result overall is achieved with leave-one-out cross-validation where the overall diagnostic accuracy is 0.92. The receiver-operated characteristic (ROC) curve for this model is shown in Fig 2C.

### Feature extraction

While the cross-validation results presented above indicate that the model generalises well with respect to the database, it is, however, the generalisation properties of the model with respect to a wider range of dermoscopic images that are paramount. Unfortunately creating a model using 125 features from a database of up to only 249 images is likely to suffer from the problem of *overfitting*. Although it may appear paradoxical to assume that the generalisation properties of the model may suffer when tested on a wider range of images (given that its generalisation performance is maximised with approximately 125 features with respect to the cohort under investigation) it is likely that the model will perform better with the dataset at hand in comparison with a wider range of lesions. This may be due to the potential similarity of lesions in the dataset: for example, images are all obtained with the same camera using the same settings, and the dataset may include multiple lesions obtained from the same patient.

Although the generalisation properties of the model are likely to improve with a feature extraction procedure that reduces the risk of overfitting, there exists the possibility that some model accuracy will be lost. However, if model accuracy is retained with a smaller number of features, then the overall performance of the model should be enhanced.

There exist a large number of different possible feature extraction methods [38]. The major alternatives lay between linear and non-linear approaches: for example, linear discriminant analysis (a supervised method) and kernel-principal component analysis (where a kernel must be chosen and its free parameters optimised). Other methods include, for example, independent component analysis, Isomap, autoencoders (within the framework of neural networks), factor analysis and non-negative matrix factorisation [38]. Ultimately, the best choice of feature extraction methodology is data-driven; that is, there is generally no *a-priori* method best-suited to all possible datasets.

### Principal component analysis

Here the feature extraction method known as principal component analysis (PCA) is applied [39]. Linear PCA is a suitable choice given the frequent occurrence of linear relationships in the feature set–either between different levels of decomposition for a particular statistical measure; or between different statistical measures at a particular level of wavelet decomposition. PCA is a widely-used, unsupervised, robust and computationally simple methodology that maintains as much data variance–given its linear constraint–as possible. Interestingly, and although clearly not the last word on the topic, in a recent study comparing a wide range of non-linear dimension reduction techniques on natural and artificial data sets, it was found that the more complex models–including, for example, Isomap and kernel-PCA–were often incapable of outperforming PCA [40].

With respect to PCA, choosing a value for the single free parameter determines how many principal components are kept, and this choice is facilitated by inspection of the resultant eigenvalue curve. The performance of the classifier is described below and is determined with respect to variation in the number of principal components utilised, where the range investigated is informed by inspection of the eigenvalue curve. Although the feature set generated by PCA does not have any physical interpretation, this is less of a problem here since the original features represent statistical measures of wavelet coefficients at particular levels of decomposition and sub-banding–none of which have any readily identifiable physical interpretation.

The singular value decomposition of the normalised (zero mean and unit variance) training data matrix Xof $X^{|}$ with dimensions p×q is given by

$$W\cdot D\cdot V^{T}$$

where p are the number of training samples, *q* are the number of features, *D* is a diagonal matrix with elements *d*_1…*q*_ corresponding to the square roots of the eigenvalues of ***$X∙X^{T}$***, and where the columms of Vare the eigenvectors of $X^{T}∙X$(where the superscript *T* denotes the transpose). The principal components *PC*_*train*_ of *X* are given by

$$PC_{train}=X\cdot V$$

Feature extraction corresponds to taking the first *n* of *q* columns of *PC*_*train*_

For the purposes of cross-validation, the standardised test data matrix *Y* with dimensions *r*×*q* r×qis obtained from the data matrix $Y^{|}$by subtracting the mean and dividing by the standard deviation of the training data. *Y* is then transformed to the othogonal coordinate system *PC*_*test*_ given by

$${PC}_{test}=Y\cdot V$$

The first *n* of *q* columns of *PC*_*test*_ are used as appropriate input for the SVM. For individual lesion assessment, the same operations are performed with the data matrix *Y* reducing to a row vector with dimensions 1×*q*.

**Fig 3:**
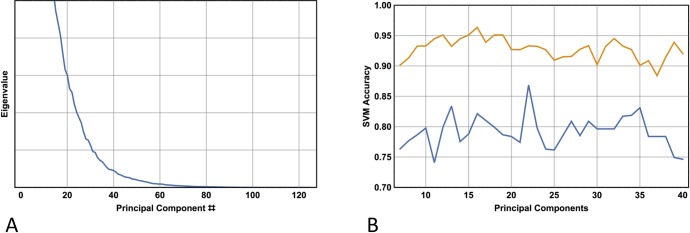
Principal component analysis. **(A)** Plot of the sorted eigenvalues of the covariance matrix associated with the data matrix ***X.***Note that the eigenvalues begin to decay more slowly at about n= 30. **(B)** SVM sensitivity (top curve) and specificity as a function of the number of principal components used in the feature reduction procedure. Note the best result overall occurs utilising 22 principal components, but the best sensitivity occurs with 16 principal components.

Fig 3A shows a plot of the sorted eigenvalues corresponding to their associated principal component, obtained from the singular value decomposition of the standardised data matrix desribed above (here the whole database is used, thus the data matrix *X* has dimensions 250 x 125; the latter value corresponds to the first 125 native features determined utilising the ReliefF algorithm). Note that the eigenvalues begin to decay more slowly for values of *n* around 25, indicating that the first 20–30 principal components can be taken with minimal information loss. Indeed, using the SVM as the image classifier, Fig 3B shows the results of 10-fold cross validation for *n* with values ranging between 7 and 40. With 22 features (principal components), the model achieves a 10-fold cross validation sensitivity of 0.93 and a specificity of 0.87.

Inspection of the top panel of Fig 2B reveals that taking the first native 22 features yields a 10-fold cross-validation sensitivity and specificity in the mid 80s and high 60s respectively. This result indicates, as expected, that feature extraction is a superior method in comparison with a feature selection method that simply takes the first 22 features that best separate the classes. On the other hand, the feature extraction method utilising 22 features (Fig 3B) yields the same result as 10-fold cross validation utilising 125 features (Fig 2B, top left panel), (accuracy 0.90 v 0.90 respectively), and exhibits slightly diminished performance compared with the best leave-one out cross validation utilising 125 features and an optimised value of C(accuracy 0.90 v 0.92 respectively; see Table 2).

**Table 2:** SVM cross-validation error rates.

**C**	**1**	**15**	**30**	**35**	**40**	**50**
**Fold**						
**10**	0.149	0.095	0.088	0.086	0.088	0.091
**25**	0.143	0.092	0.081	0.082	0.083	0.085
**250**	0.144	0.096	0.076	0.080	0.080	0.084

Mean values of error rates for the SVM following 50 replications for different levels of *N*-fold cross-validation and values of ***C***. Note that the optimal value of Cis around 30 independent of level of cross-validation and that the model improves with a larger training set.

## Discussion

This model is not the first to use the wavelet decomposition of image data for the purpose of classifying pigmented lesions, but it is likely to be the first that utilises the statistical properties of wavelet coefficients to classify lesions requiring excision from those not requiring excision. It is shown that the model, utilising the SVM as the classifier, and with only 22 orthogonal features, can achieve 10-fold cross-validation sensitivities and specificities of 0.93 and 0.87, yielding a diagnostic-odds ratio of 247. A previous meta-analyses from a total of 13 studies, where all metrics relating to the accuracy of melanocytic lesion diagnosis relied on cross-validation, revealed a diagnostic odds ratio of 15.9 [41]. Although the results presented here improves on this latter result by a considerable margin, it should be noted that the meta-analyses desribe the performance of diagnostic algorithms, not decision-making algorithms. From the clinical perspective, the reduced specificities in the diagnostic models are not surprising or necessarily problematic given the inherent difficulties in distinguishing melanoma from atypical naevi. However, the large diagnostic-odds ratio obtained with respect to the decision-making algorithm is likely to give the clinician more confidence–the output simply replicates the decision-making of an expert dermatologist with high accuracy. In contrast with diagnostic algorithms, the clinician will not need to be as vigilant regarding the possibility of false negatives.

A limitation of the present study is the relatively low number of training images; certainly a much larger training set will be required before the algorithm could be implemented in patient management. Another limitation is more general: a problem for any machine learning algorithm attempting to reproduce human classification expertise may reside in its training set labels–there is potential for a lack of consistency in these labels due to non-concordance in the decision-making of human experts. If an algorithm is trained with conflicting examples, its performance may be compromised. As noted in the Introduction, this is a potential problem when classifying pigmented lesions based on histopathological diagnoses. Yet clinical decision-making will also suffer from the same limitation. By restricting the human classifier to one renowned expert, consistency can be achieved at the expense of potential bias. On the other hand, using a majority rule among multiple experts’ decisions can mitigate bias at the expense of consistency. The trade-off between consistency and bias and its impact on the veracity of training data is thus an important consideration. Reassuringly, in the training set reported here, all lesions were classified by only a small number of dermatologists, and all the dermatologists received their specialist training under the same conditions.

The relatively small number of features used in the model– 22 –suggests that over-fitting is unlikely to be a problem, thus, from the perspective of model complexity, its generalisation properties will not be compromised. Importantly, however, by virtue of its modularity and flexibility, the analysis pipeline will permit the model to evolve, and thus improve its generalisation capabilities as new training data are acquired. Its major modular components are: (i) *the choice of wavelet function*; (ii) *the level of wavelet decomposition*; (iii) *the statistal measures derived from the wavelet coefficients;* (iv) *the method of ranking the utility of features in the classification task*; (v) *the feature selection methodology*; (vi) *the feature extraction methodology*; and finally, (vii) *the choice of artificial intelligence algorithm*. Any or all of the modular choices used in the current implementation can be substituted with alternatives, and tested, as new training data are acquired. Interestingly, as noted in the Results section, the performance of the model in 10-fold cross validation reached a maximum at around 125 features. This observation suggests that increasing the number of training set examples may not necessarily lead to further increases in the optimal number of native features required to best separate the classes. If ~125 native features optimise cross-validation performance with two thousand training samples, then it may not be necessary to extract a reduced number of principal components, as overfitting these data may not then be an issue.

Consider the implementation of the algorithm in the clinical setting. Although the findings are based on a relatively small training sample, the results are encouraging. By appoximating the performance of expert dermatologists, the high sensitivities indicate that the model shows potential in not compromising melanoma detection, while the high specificities suggest the model may be of assistance in reducing the number of unnecessary excisions, thus lowering the NNT of practitioners in general. As defined elsewhere [21], the algorithm can act as a decision support tool; more specifically, it is envisioned that if either the clinician, on clinical grounds, *and*/*or* the algorithm, based purely on morphologic grounds, indicates that the lesion should be excised then it should be excised. The lesion should only not be excised if both clinician and algorithm agree that the lesion does not require excision.

Although this strategy appears simple and effective, there are problems associated with the use of automated tools in pigmented lesion assessment. While size, site and history of a given lesion can yield information that may determine a management decision irrespective of the morphology *per se*, it is *context* that poses the major problem for any automated assessmnet tool [42]. For example, a single darkly pigmented melanocytic lesion present on the posterior leg in a female red-head should probably be excised, irrespective of its morphology. Yet if the lesion appears to be a benign compound naevus, then the decision-making algorithm is likely to recommend no excision. On the other hand, some patients can exhibit multiple atypical pigmented lesions, particularly if there is a history of excessive sun exposure. The majority of these lesions, however, are usually benign junctional naevi. The decion-making algorithm described here may suggest, wrongly, that *all* lesions should be excised. Yet all is not lost: in the case of the red head the algorithmic decision not to excise should be ignored; while in the case of patients with multiple atypical naevi the algorithm may have utility–given its probabilistic output–in detecting the ‘*ugly duckling*’; that is, the lesion that is most likely melanoma [43].

**Fig 4:**
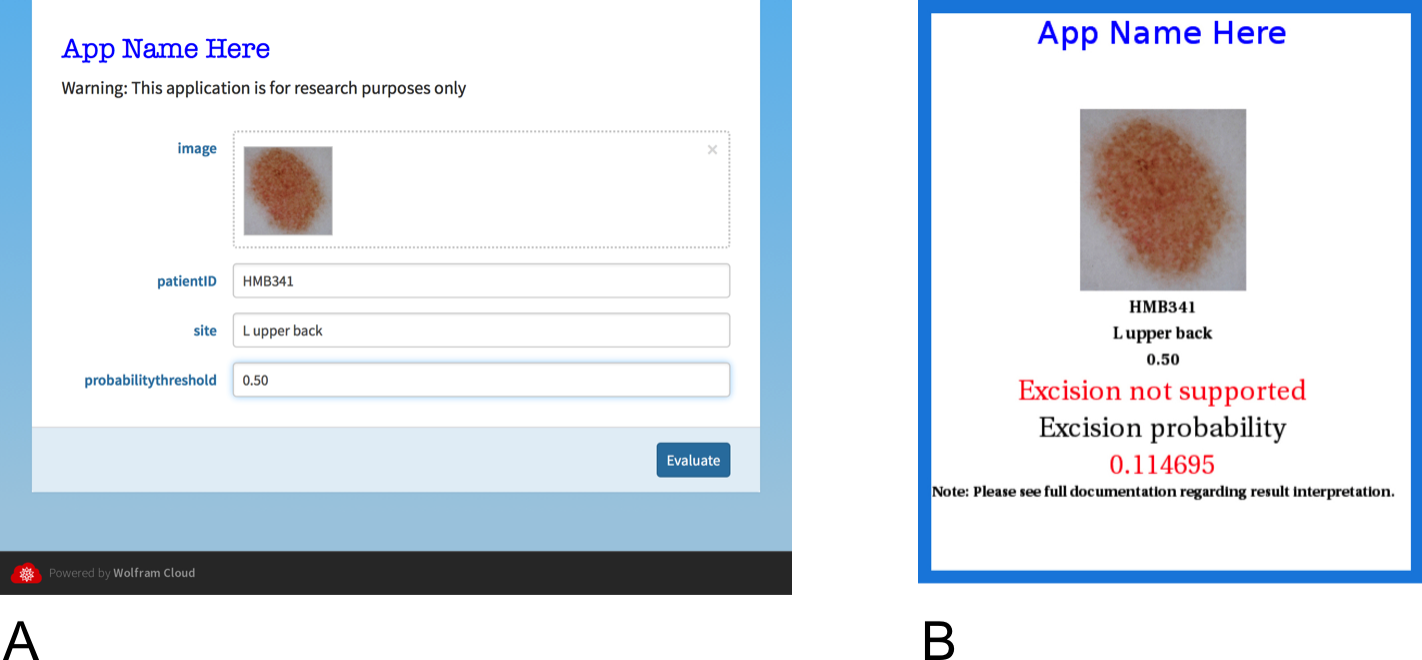
Screenshots of the application as it appears on a smart device. **(A)** Data entry page showing the image to be analysed (which can be imported directly using the smart device’s camera, or from a file by dragging or using a file directory), the patients ID code, the site of the lesion, and the probability threshold (which is by default set to 0.50). **(B)** The output page is self-explanatory. (Reproduced with permission [23]).

Finally, a smart device application has been developed, based on the Wolfram platform [23], which allows clinicians to easily utilise the algorithm in the clinical setting (Fig 4A). Using, for example, the *Handyscope*^*TM*^ attachment to an *iPhone*^*TM*^, the clinician is able to take high quality polarised images of pigmented lesions and immediately input them to the algorithm, where the computations take place in the cloud. Alternatively, images can be saved on the smart device or transferred to a computer and submitted for analysis at any time. Results are displayed on the smart device within 30 to 45 seconds and can be given as either a binary output (excise; do not excise) or as a probability of class membership (Fig 4B). It is planned to introduce the algorithm as a research tool where the aims are twofold: first, to formally assess the application’s clinical acceptability; and second, to utilise the images obtained to periodically expand the training set, update the algorithm, and re-evaluate its performance.

### Hardware and software

All computations were performed on a MacBook Pro, running at 2.5GHz and with 8GB RAM (Apple Corporation, California, USA). All computations were performed using either *Mathematica V10* (Wolfram Research, Illinois, USA) or the *R* platform: (R Core Team (2014). R: A language and environment for statistical computing. R Foundation for Statistical Computing, Vienna, Austria. URL: http://www.R-project.org/).

## Supporting information

S1 File noexcision. (CSV)

S2 File excision. (CSV)
